# Advance care planning as perceived by marginalized populations: Willing to engage and facing obstacles

**DOI:** 10.1371/journal.pone.0301426

**Published:** 2024-04-01

**Authors:** Shigeko (Seiko) Izumi, Ellen Garcia, Andrew Kualaau, Danetta E. Sloan, Susan DeSanto-Madeya, Carey Candrian, Elizabeth Anderson, Justin Sanders

**Affiliations:** 1 School of Nursing, Oregon Health & Science University, Portland, Oregon, United States of America; 2 Bloomberg School of Public Health, Johns Hopkins University, Baltimore, Maryland, United States of America; 3 College of Nursing, University of Rhode Island, Providence, Rhode Island, United States of America; 4 School of Medicine, University of Colorado, Aurora, Colorado, United States of America; 5 Pacific Institute for Research and Evaluation, Louisville, Kentucky, United States of America; 6 Department of Family Medicine, McGill University, Montreal, Quebec, Canada; Emory University, School of Public Health, UNITED STATES

## Abstract

**Background:**

Health disparities exist in end-of-life (EOL) care. Individuals and communities that are marginalized due to their race, ethnicity, income, geographic location, language, or cultural background experience systemic barriers to access and receive lower quality EOL care. Advance care planning (ACP) prepares patients and their caregivers for EOL decision-making for the purpose of promoting high-quality EOL care. Low engagement in ACP among marginalized populations is thought to have contributed to disparity in EOL care. To advance health equity and deliver care that aligns with the goals and values of each individual, there is a need to improve ACP for marginalized populations.

**Aim:**

To describe how patients from marginalized populations experience and perceive ACP.

**Methods:**

We used an interpretive phenomenological approach with semi-structured qualitative interviews. Participants were recruited from four primary care clinics and one nursing home in a US Pacific Northwest city. Thirty patients from marginalized populations with serious illness participated in individual interviews between January and December 2021. Participants were asked to describe their experiences and perceptions about ACP during the interviews.

**Results:**

The mean age of 30 participants was 69.5; 19 (63%) were women; 12 (40%) identified as Asian/Pacific Islanders, 10 (33%) as Black; and 9 (30%) were non-native English speakers. Our three key findings were: 1) patients from marginalized populations are willing to engage in ACP; 2) there were multiple obstacles to engaging in ACP; and 3) meaningful ACP conversations could happen when clinicians listen. Although participants from marginalized populations were willing to engage in ACP, a fragmented and restrictive healthcare system and clinicians’ biased behaviors or lack of interest in knowing their patients were obstacles. Participants who felt their clinicians took time and listened were encouraged to engage in ACP.

**Conclusion:**

Patients from marginalized populations are willing to engage in ACP conversations despite a common belief otherwise. However, obstacles to meaningful ACP conversations with healthcare providers exist. Clinicians need to be aware of these obstacles and *listen* to build trust and engage marginalized patients in mutually meaningful ACP conversations.

## Introduction

Advance care planning (ACP) helps people prepare for future healthcare decisions and receive care that aligns with their goals and values [1–3]. Although the concept of ACP is broadly accepted and is currently being implemented in healthcare practices, ACP among marginalized populations remains low [4–10]. Marginalized populations are described as those who live on the margins of mainstream society [11] and “are peripheralized on the basis of their identities, associations, experiences, and environments” [12, p.25]. They may be seen as being “cast aside,” “hidden,” or “stigmatized” in society, thus marginalized groups often lack access to services and report mistrust in systems [12]. People are marginalized based on their race, ethnicity, gender identity, sexual orientation, income, geographic location, age, physical ability, language or religious or cultural background [11, 12]. Members of marginalized populations, such as minoritized racial and ethnic groups [4, 10, 13–18], persons in the LGBTQ community [6, 7, 19–21], and those with low socioeconomic status, health literacy, or low English proficiency [14, 22–28] engage less in ACP and experience disparities in care. Some researchers have attributed lower rates of ACP among marginalized populations to patient factors such as religiosity, culture, and mistrust [29–34]. Literature also indicates that clinicians have implicit biases and may communicate differently or avoid ACP conversations with patients from marginalized populations [35–41].

Although attention to ACP for different racial or ethnic groups is growing [6, 9, 18, 42–49], little is known about effective ways to facilitate and engage patients from marginalized populations in ACP [50]. In addition, while the effort to explore different views about ACP by different groups is valid, we question the notion of developing different ACP approaches for different racial or ethnic groups, because we believe that each person should be respected as a unique individual and should not be categorized simply by race and ethnicity. White persons could be marginalized due to their economic status, gender, or sexual orientation. Affluent Black or Latino men may experience marginalization differently from Black or Latina women in poverty. Discrimination and marginalization occur in intersections of race, ethnicity, sexual orientation, gender identity, disability, class and other forms [51, 52]. There is a need to explore approaches to ACP that are meaningful, acceptable, and equitable to each individual beyond stereotypes [53, 54]. The purpose of this study is to describe how patients from diverse marginalized populations experience and perceive ACP as a step to develop an inclusive equitable ACP for all.

## Methods

This interpretive phenomenological study [55] using semi-structured in-depth interviews was conducted from January through December 2021. Interpretive phenomenology derives from the Heideggerian philosophy which posits that an individual’s world-view is constructed by their own lived experiences [55]. Researchers using the interpretive phenomenological approach explore the participants’ perspectives about a phenomenon by co-interpreting the participants’ lived experiences that form their world-view [55, 56]. The interpretive phenomenological approach was particularly appropriate to understand how participants with different background perceived or experienced ACP based on their lived experiences. This study was approved by the Providence Health System Institutional Review Board (Study # 2019000537). The reporting in this paper adheres to the Standards for Reporting Qualitative Research (SRQR) [57].

### Study population and sample

We purposefully recruited patients from various marginalized populations who were living with serious illness, as they were likely to have considered ACP. We used the presence of one or more of the following diagnoses or conditions to identify participants with serious illness [58]: cancer, renal failure, advanced liver disease, diabetes with severe complications, severe lung disease, hospitalization for heart failure, and/or functional limitations receiving assistance with activities of daily living. We used a purposive sampling method to include participants from diverse marginalized groups. Grounded in the literature, our criteria for marginalized population included those who self-identified as a racial/ethnic minority (e.g., Black/African, Asian, indigenous); persons with Medicaid eligibility; persons experiencing housing instability; individuals whose preferred language was not English; and/or self-identified as being lesbian, gay, bisexual, trans, or queer [11]. The eligibility for Medicaid was used as an indication of lower economic status in the electronic health records (EHR) at recruitment sites. Housing instability was identified through patient reports of experiencing houselessness, sleeping in a car, couch-surfing at a friends’ house, or if their contact address was a local homeless shelter as documented in an EHR. Gender or sexual orientation minority status was not routinely documented in EHR. If patient’s EHR included some notes or clinicians knew the patients, they were referred to the researchers and the researchers verified status as part of the recruitment procedure.

We sampled from four primary care clinics and one nursing home that served diverse inner-city communities in the Pacific Northwest. Clinicians at study sites were asked to identify patients who met our study criteria and to refer interested patients to the researchers. Researchers then contacted potential participants by telephone, described the study, confirmed inclusion criteria were met, and obtained verbal consent for an interview. During the consent process, we told participants that they would receive a $50 gift card for a local grocery store at the end of the interview in appreciation of their time. Interviews were scheduled by participants’ preference (i.e., in-person, via telephone, video call) following state COVID-19 guidelines at the time of interviews.

### Data collection

Semi-structured interviews were conducted by an experienced qualitative researcher (SI), a registered nurse with formal training as an ACP facilitator, using an interview guide developed with the research team. The guide included questions about whether participants had planned for future healthcare. If so, the interviewer asked about their preferences and experiences of planning. If not, the interviewer asked goals/values questions commonly used in facilitated ACP conversations and asked about the participant’s thoughts around having similar conversations with their families and healthcare providers.

The mean interview time was 38 minutes (range 19–89 minutes). Interviews were audio-recorded and transcribed verbatim by a HIPAA compliant professional transcription service. Returned transcriptions were reviewed for accuracy and de-identified by replacing identifiable information such as personal or organization names with random codes by a research assistant. Adequacy of sampling size in interpretive phenomenology depends on the depth and richness of the data [56]. Three key themes of ACP experiences were identified by the 24^th^ interview. Further interviews added more variation, but no new recurring themes. We concluded data collection after 30 interviews.

### Analysis

We analyzed the transcripts using an interpretive phenomenological approach [55, 59]. Three researchers (SI, EG, AK) first individually read the transcripts and inductively coded exemplary perspectives or experiences related to ACP. We met regularly comparing codes and exemplary quotations from the interview data looking for the commonalities, differences, and patterns across participant experiences and meaning, and discussed until achieving a shared understanding about the participants’ experiences and perceptions about ACP. Following the interpretive phenomenology tradition, data were interpreted through the lenses constructed by each researcher’s lived experiences [60]. Before and throughout the analysis, researchers from different cultural backgrounds and positions (e.g., Asian, White, Native Hawaiian, sexual orientation minority, immigrant, English as a second language, nurse, professor, PhD student), reflected on how their own experiences informed or potentially biased their interpretations. To enhance the transferability, authenticity and trustworthiness of these researchers’ interpretations of the data, we invited external researchers (CC, DS, EA, SDM, JS) with expertise in ACP among diverse patient populations to review our preliminary findings.

## Findings

Of 49 patients referred, 30 agreed to participate. The average age of participants was 69.5 (range 36–93); approximately two third were female; the most represented racial groups were Asian and Black. Having multiple diagnosis of chronic conditions was common (Table 1).

**Table 1:** Demographic information about participants (n = 30).

Age Mean (range)		69.5 (36–93)
Gender	Female	19 (63%)
	Male	11 (37%)
Underrepresented sexual orientation (self-identified)		2 (7%)
Race	Asian/Pacific Islander	12 (40%)
	Black	10 (33%)
	Native American	2 (7%)
	White	6 (20%)
Latino		2 (7%)
Non English native speakers		9 (30%)
Interviewed with an interpreter		6 (20%)
Low socioeconomic status (i.e., Medicaid recipient, houseless)		6 (20%)
Types of serious illness		
Diabetes Mellitus		12 (40%)
Cardiovascular Disease		9 (30%)
Chronic Kidney Disease		9 (30%)
Congestive Heart Failure		8 (27%)
Cancer		8 (27%)
Mental Disorder		6 (20%)
Liver Disease		4 (13%)
Chronic Obstructive Pulmonary Disease		2 (7%)

We identified three interrelated themes: 1) patients from marginalized populations were willing to engage in ACP; 2) there were multiple obstacles to engaging in ACP; and 3) meaningful ACP happened when clinicians were engaged and listen.

### Patients from marginalized populations are willing to engage in ACP

More than one third of participants had previously engaged in ACP conversations with their family members and/or healthcare providers. Some were clear and ready to discuss with providers what kind of care they would want to receive at EOL, others were not clear about what EOL care they wanted. All participants discussed their goals, values, and ACP when asked by the interviewer.

Past exposure to EOL care or serious illness experience acted as a key difference between those who had or were ready to have ACP conversations and those open to conversations but not ready to express their care preference. Past EOL experiences included being seriously ill and hospitalized, caring for dying family members or friends, or seeing people near EOL in their work. Because of these experiences, these participants had a sense of what to expect in EOL care, and that led them to know “what I want (or don’t want)” in such situations.

I don’t want any of that [chest compressions, etc.]. When my wife passed away, her heart stopped right there [in a clinic]. And they tried to revive her. I was told that, very often in doing these compressions, they break the breast-bone, and all sorts of things. I don’t think I want any of that.

(ID 5: *90 yo*, *Asian male*)

A Native American participant took care of her children’s father when he died and worked as a paramedic. She said, “A lot of times, you get into hospitals, they are primarily run by Whites who don’t have an understanding of our culture. So, you don’t want them to make the decision for you, but that’s what’ll happen if you don’t have it [your wishes] on paper. (ID32: 58 yo. Native American female)” For her and some other participants, their assumption that healthcare providers would not understand their goals and cultural values motivated ACP. She wrote her care preferences on paper and instructed her adult children about the paper, hoping that her cultural preferences will be respected in a White-dominant healthcare system.

In contrast, participants without EOL care experience had difficulty imagining their EOL care situation and did not have a clear idea what they wanted. Their responses regarding ACP were vague—“do everything,” “go natural”, or “follow what my doctors tell me to do.” Some participants without EOL care experience responded “It is in God’s hand” or “my family will make that decision for me,” leaving the decision-making to someone else. Although participants without past experiences were less clear about their EOL care preferences, they all engaged in ACP conversations exploring their own life goals and values with the interviewer.

### Multiple obstacles to engage in ACP

While the participants were willing to engage in ACP, we identified a range of obstacles keeping participants from having ACP conversations with healthcare professionals. These obstacles were grouped into three categories: patient characteristics, clinicians’ behaviors, and healthcare system (Table 2).

**Table 2:** Obstacles participants faced to engage in advance care planning.

Patient characteristics	Clinicians’ behaviors	Healthcare system
Limited knowledge about end-of-life care due to lack of past end-of-life care experience Cultural values and belief about authority of healthcare professionals	Lack of understanding of patient situation Seeming lack of interest in knowing the patients Disrespectful behaviors based on biases Indication or subtle signs of bias or stereotyping Not listening to patient	Healthcare plan coverage and rules Clinician turn over Cost or lack of access to clinicians they trust Inconsistent documentation Interpreter availability Limited time for visit

#### Patient characteristics

As described in the prior section, patients not having past EOL care experiences could be an obstacle for ACP conversations, because they were not clear about what to expect or what needed to be discussed. When the interviewer explained what might happen and inquired about their goals and values, the participants were willing to engage in ACP. Some participants, mostly first-generation immigrants from Asia, had a cultural belief that healthcare providers have expertise and authority to make EOL care decisions that superseded their personal preferences.

Participant: If the hospital says that I have to have it [tube feeding], I guess I have it.
If the doctor recommends it, then I will follow the doctor.
Interviewer: If you got to make the decisions, is this something you want to have?
Participant: No, I don’t want tube feedings.
Interviewer: How about a breathing machine?
Particiant: Well, if you want to live, that’s my only option, right?

Interviewer: If the doctor says it’s really up to you, do you want it?

Participant: No, I’m quite old; if that’s the case, I would rather just die naturally. (ID 26: 83 yo. Asian male, living in a nursing home. Interviewed with an interpreter)

However, most participants (including other Asian immigrants) who had past EOL care experiences understood there would be a time when healthcare providers would want to know patients’ preferences for EOL care.

Another participant (ID3 65 yo Black female) described her beliefs, “I was raised very religious. We only have one maker, and He’s the one who makes all decisions. We do have a hand in it, but He’s the one who makes the final decision.” Yet, she talked about her ACP and expressed care she wants to receive at EOL as “My position on that [EOL care] depends on quality of life. If you have no quality of life left, you don’t need to be put on life support. Just make yourself comfortable, talk to the family about how to go about making peace with the Lord.” This participant used to work as a certified nurse aid in nursing homes and had seen EOL care. For this participant, it was apparent that religous beliefs and EOL care preferences could coexist without being a barrier to ACP. These data indicate that lack of EOL care experiences is a key obstacle stopping patients from ACP conversations, and patients’ religious or cultural beliefs (such as trusting healthcare professionals’ authority, cultural value of conformity) are lesser factors determining patients willingness to engage in ACP conversations.

#### Clinicians’ behaviors

Clinicians’ behaviors that appear to reflect bias, stereotyping, or lack of understanding of participants’ situation appeared to act as obstacles to ACP conversations, even when participants might have been ready to engage in ACP. One participant with COPD who lived in a vehicle was not able to obtain the medication she needed, because providers seemingly did not listen to her or attempt to understand her situation.

I have to call my doctor and let them know that I need another inhaler because I am almost out. And they said it should last for a month. I am living in a van, it is cold. Because of the cold weather, I have to use it. I might be using a little bit more, but I can’t help it.”

(ID8: 54 yo, Native American female, experiencing homelessness)

When this participant went to an urgent care to get the inhaler, she was labeled as “non-compliant” and denied her medications.

A doctor at urgent care stopped all my medications. He told me that if I don’t go to take counseling for stop smoking. We were in the middle of moving at the time. I tried to explain to him, “We’re moving right now, so I can’t take time out [to attend counseling].” … He goes, “Well, then, you give me no choice.” And he stopped all my medications at the pharmacy anywhere I went.

(ID8: 54 yo, Native American female, experiencing homelessness)

This participant said she was treated this way because she is homeless and smokes, and she believed that this physician had no intention to listen or understand the circumstances of her situation, so she did not see a point of talking about ACP sharing her goals and values with providers. Several other participants described that providers had no intention or interest in understanding their lives. One Black female participant (ID 2) said “White doctors don’t get me;” describing how her physicians did not understand that she has to take care of grandchildren while recovering from a hospitalization for heart failure. Providers’ lack of interest in understanding patients’ situation made participants unwilling to have ACP conversations with the providers.

Another participant described her experiences of clinicians being disrespectful because of her race, gender, and how she looks.

If you don’t stand up for yourself, they [clinicians] will walk all over you. Since I’m fat, I’m black, and I’m female, you go in and you encounter this, ‘you lose weight.’ … I came along in an age when men thinking they were smarter than women.”

(ID 13: 82 yo, Black female)

Another Black female participant described how clinicians racially stereotyped and failed to recognize her as her father’s surrogate decision maker impacted his EOL care and her own ACP.

I was the decision maker for my father. He was bleeding, and they [hospital clinicians] stabilized him a couple of times. At one point in the middle of the night, they decided he needed to be on a respirator. My half-sister, who is probably 20–30 years younger than me, and honest to God, does not look anything like me. But sometimes *all black people look alike*. They mistook her for me. And she authorized the ventilator.… It was just awful….

(ID 22: 66 yo, Black female)

Because of this experience, this participant told the interviewer she wanted to have clear ACP to make sure what happened to her father was not repeated with her. However, her healthcare providers never engaged her in ACP.

Some participants described where they see potential signs that they might be discriminated against and how they proactively advocated for or protected themselves by not sharing their values and preferences. A participant who self identified as a lesbian shared her experience.

As I walked in his [a physician’s] office first time, I saw a diploma from XYZ University, that is known for its homophobia. So, I sat down with him. He seemed to be someone who was open person that I could have as a medical person. So I just said to him, "Okay. I need to ask you. I saw you have graduated from XYZ University. I’m a lesbian. I’m in a partnered relationship…. Is that going to be a problem for you?" He sat back and said, "I’m married and have three kids. Is that going to be a problem for you?" And I said no. He says, "Okay. We can work together."

(ID 9: 73 yo, White female, self-identified as a lesbian)

Although this participant directly confronted the provider, other LGBTQ+ persons may take the diploma as a sign of his bias and therefore may withhold disclosure of their personal goals and values or who her surrogate decision maker is.

#### Healthcare system

The complex healthcare system poses many obstacles for patients from marginalized population engaging in ACP with their healthcare providers. Loss of insurance due to loss of jobs, health organization mergers, and frequent changes in insurance rules restricted access to healthcare providers with whom they had built or could build a relationship and have ACP conversations.

When I changed insurance, they [insurance representative] said, “You can’t go to this provider” I had built a rapport with, who knew me and were watching my medications, “because you can only see this one mental health professional in our system.”

(ID 24; 70 yo, White male, experiencing homelessness and mental health issue)

One clinic where participants were recruited was a federally qualified health center affiliated with an academic medical center. A participant from this site stated:

I’d want somebody who knew me or who I had a relationship with. You used to be able to have that with your doctor, but you can’t anymore because you only get 15 minutes a visit. My doctors are all residents. They’re in and out every four years…. It doesn’t give me confidence to establish a relationship with a group of people that are running in and out like water.

(ID 22; 66 yo, Black female)

Some participants said that they usually go to urgent care and do not have a healthcare provider with whom they can build a relationship.

In a fragmented healthcare system, ACP relevant information (e.g., medical history, test results, or advance directives) are often lost, especially when participants see multiple providers. Participants said they had to submit forms to release their health information from other clinics or hospitals again and again. When earlier ACP conversations were not documented or found, participants had to start conversations anew.

Without adequate time and reliable interpreter services, patients with limited English language proficiency found it almost impossible to have nuanced ACP conversations or talk about their goals and values thoroughly.

I would like the doctor, if possible before they examine me, if they can spend maybe 20–30 minutes to have a conversation with me, asking like “what is your wish?” So, that way it triggers me to start talking to them. … But I haven’t had the chance to talk to [my doctor].

(ID 20, 67 yo, Asian male, living in a nursing home, interviewed with an interpreter)

Several participants noted how busy their providers are and identified limited time as another system obstacle preventing ACP conversations.

### ACP happens when clinicians are engaged and listen

Despite these obstacles, approximately one third of participants said they had had ACP conversations with their healthcare providers. Most of them had had long term relationships and said they trusted their providers. They often had positive experiences that fostered trust.

That’s why I love my primary care doctor I have now, because she listens to what I say…. She trusts me, I trust her, and she listens to me.… That’s how we found out that I had brain cancer. I was seeing double vision, and I told her, “Is there any way I can get an MRI just in case my cancer has traveled to my brain?” She listened to me and she put in for an MRI, so we caught it in time.

(ID 3: 65 yo, Black female)

A subgroup of participants, mostly first generation Asian immigrants, expressed respect and trust for healthcare professionals in general (e.g., “I trust people in the hospital to take care of me”). However, when the interviewer asked how they would like to have ACP conversations with their providers, many preferred to talk with providers who know them and take time to listen.

Providers engaging with patients by taking time to listen, trying to know the patients, and explaining what to expect with future care was key to building trusting relationships and setting the foundation for ACP conversations. One participant explained, “My provider has time for me. She sits and listens to me, takes notes (ID 7: 53 yo. Black female)”. All interview participants engaged in ACP conversation with the interviewer, although they had never met the interviewer before. The interviewer was a trained ACP facilitator skilled in active listening, and she had 40–60 minutes to talk with participants. Some participants appreciated the interviewer for spending the extra time to explore meaning and values. This suggests that meaningful ACP conversations with patients from marginalized populations can happen when they believe that clinicians have time and are actively listening.

## Discussion

In contrast to the common belief that patients from marginalized populations do not engage in ACP [33, 34, 40, 61], participants in this study engaged or were willing to engage in ACP conversations. However, we identified a wide range of obstacles precluding patients from marginalized populations from having ACP conversations with healthcare providers including patient beliefs, clinicians’ behaviors, and a fragmented healthcare system. Clinicians’ behaviors and attitudes towards patients played the major role for participants to determine whether they engage in ACP. That participants engaged in ACP during our interviews yet had not done so with their providers raises questions about what clinician behaviors, communication skills, or fragmented and time limiting healthcare system contribute to disparities in ACP engagement for patients from marginalized populations [39, 62].

Patient inexperience with EOL care as an obstacle in ACP is not unique to patients from marginalized populations. Findings from other studies suggest that patients in general have difficulty imagining preferences for EOL care and engaging in ACP if they do not have past EOL care experiences [46, 63]. This is an obstacle intrinsic to patients, yet clinicians are responsible to educate patients about illness and what to expect as part of ACP preparing them for future decision-making [64]. What makes a lack of EOL care experiences a unique challenge for marginalized groups is that clinicians tend to stereotype and assume these communities do not engage in ACP due to religious, cultural, and/or personal beliefs, not because they do not know what to expect in EOL care. Clinicians may exclude them from ACP based on stereotypes [39] (e.g., “she is Black, she must be religious, believe in God, and not talk about ACP”) instead of having the conversation and educating them about what to expect.

Participants in this study were marginalized for various reasons (e.g., “because they were Black, Native American, immigrant, lesbian, homeless, not speak English well”) and likely to be seen by healthcare providers as *others* who are different from mainstream people one way or another. Regardless of different reasons for marginalization, all participants without EOL care experiences who initially did not articulate their goals and values still engaged in ACP conversation with the interviewer. This finding implies the possibility of an ACP approach that could be acceptable by many people including those from a broad range of marginalized populations. Potential strategies to overcome clinicians stereotyping patients from marginalized population and facilitate ACP with all patients include asking about past EOL care experiences and using experiences to reflect on their goals, values, and future care [63]. If patients have not had such past experiences, clinicians can provide guidance about what to expect followed by exploration of their life goals and values [65].

This study’s findings challenge the notion of people from marginalized groups not trusting healthcare providers to have ACP conversations [30, 39, 66–68]. Some participants expressed having trusting relationships with their providers and had meaningful ACP conversations. On the other hand, if clinicians’ behaviors suggested biases or disinterest, participants thought that the clinicians would not listen, and they did not trust or engage in ACP. Whether participants from marginalized populations trusted their providers was based on how the individual clinician they encountered behaved. Some participants tried to engage clinicians by speaking up for themselves even risking vulnerability (e.g., disclosing their same sex marriage) and watched how clinicians responded to assess their level of safety with the clinician. Clinicians need to be responsible to build relationships with their patients and earn their trust. Our participants described clinicians they trusted as engaged, paid attention, and actively listened to learn who they are Johnson et al. [69] suggested clinicians ask and find out who the patient is using cultural humility before talking about their goals, values, and preferences.

Participants engaged in ACP conversations with the interviewer whom they had just met without an established trusting relationship. One potential interpretation is that ACP conversations outside of the healthcare setting facilitated by someone neutral not directly involved in their care may offer a safe environment for patients to contemplate and engage in ACP. Because the interviewer was a nurse trained to facilitate ACP conversations, it also could be that ACP facilitation skills may be useful to engage patients from marginalized groups in ACP even when there is no long-term relationship. Some ACP facilitation skills (e.g., silence, active listening) and phrases (e.g., “tell me more”) could be useful to guide clinicians’ efforts to listen to know who the person is, and to start building trust. Clinicians’ behaviors reflecting their cultural humility, communication skills, and interest and awareness of patient experiences could facilitate meaningful ACP conversations with patients from all backgrounds and foster trust.

The current healthcare system poses other obstacles limiting patient access to trusting providers and sufficient time to have meaningful conversations. Although more effort is needed to eliminate these system obstacles, clinicians can mitigate them and facilitate meaningful ACP by being aware of their own biases and the obstacles patients may be experiencing, and intentionally listening and learning who they are. Securing time to listen to patients could be a challenge. Exploring the ways to integrate small parts of ACP conversations over the course of multiple visits may be a strategy to mitigate time constraints.

This study has limitations. Participants were from a limited geographic area. Information about sexual orientation and gender identity were often missing or inaccurate in health records, and Medicaid or self-disclosed information about socioeconomic status were not necessarily indications of marginalized status. Because we defined marginalized population broadly, the number of participants representing each subgroup was small, and our findings do not represent the perspective of any specific subgroup. However, purposive sampling resulted in a diverse group of study participants whose shared ACP experiences might be transferable to those of other and marginalized persons. A future study that describes ACP interactions from both patient and clinician perspectives in dyads could further advance our understanding of clinician behaviors or communication skills that facilitate patient engagement in ACP including those from marginalized populations.

## Conclusion

Contrary to beliefs that those from marginalized populations do not wish to engage in ACP, our participants were willing to do so. However, many obstacles impede marginalized individuals from taking part in ACP conversations with healthcare providers. Findings from this study suggest that adding questions about past EOL care experiences and emphasizing active listening may enhance existing ACP approaches, increasing inclusivity and alleviating disparity in ACP. Clinicians should be aware of these obstacles, including their own biases and behaviors, and learn how to listen to build trusting relationships that serve as a foundation for ACP.

## Supporting information

S1 Checklist SRQR checklist. (DOCX)
